# Long-Term Follow-Up after Testicular Torsion: Prospective Evaluation of Endocrine and Exocrine Testicular Function, Fertility, Oxidative Stress and Erectile Function

**DOI:** 10.3390/jcm11216507

**Published:** 2022-11-02

**Authors:** Peter Törzsök, Christopher Steiner, Maximilian Pallauf, Michael Abenhardt, Ljiljana Milinovic, Bethseba Plank, Alena Rückl, Manuela Sieberer, Lukas Lusuardi, Susanne Deininger

**Affiliations:** 1 Department of Urology and Andrology, Paracelsus Medical University, Salzburg University Hospital, 5020 Salzburg, Austria; 2 Department of Urology, The James Buchanan Brady Urological Institute, Johns Hopkins University School of Medicine, Baltimore, MD 21211, USA; 3 Department of Pathology, Paracelsus Medical University, Salzburg University Hospital, 5020 Salzburg, Austria

**Keywords:** fertility, oxidative stress, semen analysis, spermatic cord torsion, testicular torsion, testosterone

## Abstract

Background: This study investigates endocrine and exocrine testicular function, oxidative stress (OS) in semen, and erectile function in patients who underwent surgery for suspected testicular torsion (TT). Methods: We evaluated 49 patients over a mean follow-up of 101 months: *n* = 25 patients treated with surgical exploration, *n* = 20 patients treated with detorsion, and *n* = 4 treated with orchiectomy. We performed semen analysis including Male infertility Oxidative System (MyOxSIS) analysis, physical examination, and evaluation of endocrine and erectile function. Results: OS, erectile function and spermiogram categories did not differ significantly between the groups. The interval from the onset of symptoms to surgery differed significantly between groups (*p* < 0.001). Preservation of the testes was associated with a higher round cell count (*p* = 0.002) and follicle stimulating hormone (FSH, *p* = 0.003). OS showed a significant positive correlation with the spermiogram category (0.337; *p* = 0.022). A negative correlation was observed between OS and age (*p* = 0.033), sperm concentration (*p* < 0.001) and total sperm count (*p* = 0.006). Conclusions: Endocrine, exocrine and erectile function are not significantly affected by TT in the long term. Orchiectomy results in elevated FSH and a lower round cell count compared to preservation of the testis.

## 1. Introduction

Testicular torsion (TT), usually a spontaneous twisting of the testis around the spermatic cord causing subsequent ischemia, affects 1/4000 men younger than 25 years of age [1,2]. It is a urological emergency. A suspected TT calls for immediate surgical exploration with detorsion and fixation or removal of the affected testis [3]. In order to avoid severe testis injury, surgery should be performed within six to eight hours, at the latest, after the onset of symptoms [4,5].

Experimental animal studies showed that unilateral TT is capable of reducing fertility by damaging the blood–testis barrier (BTB). However, this only occurs when the twisted testicle is left in place [6]. Furthermore, injuries to the BTB can provoke an immune response, damaging both testis [4,7].

Permanent impairment of testicular function is thought to be caused by ischemia at the time of TT and by oxidative stress (OS) following detorsion [8]. Reperfusion hyperemia after detorsion of the testis causes the production of reactive oxygen species (ROS). ROS may provoke testicular DNA impairment, which may culminate in germinal cell apoptosis [9]. OS is regarded as one of the main elements of male infertility [10,11,12] and is negatively correlated with sperm concentration and motility [13,14].

The primary endocrine function of the testis, which is the production of testosterone, appears to remain unaffected by TT [15,16]. However, reduced testosterone and elevated follicle-stimulating hormone (FSH) and luteinizing hormone (LH) levels can be found after TT [17]. Notably, elevated testosterone levels were also detected after TT compared to controls [4].

Impairment of exocrine testicular function has been described in various studies, which also report altered sperm morphology and decreased sperm motility and sperm count [4,16,17,18]. Although the clinical effect of impaired semen quality has not been specifically addressed in these studies, pregnancy rates were similar in comparisons of men after TT and the average male population [19].

We lack a sufficient body of long-term data on the impact of testicular function after TT on erectile function [20,21]. Moreover, to our knowledge no study has assessed OS after TT in the long term.

The present study includes a long-term follow-up of endocrine and exocrine testicular function, OS in semen, and erectile function in patients who underwent surgery for suspected TT.

## 2. Methods

The data of patients who underwent surgery for suspected testicular torsion between 1 January 1999 and 1 September 2020 at the University Clinic of Urology and Andrology, Salzburg, were analyzed. Three hundred and nineteen men were identified and contacted between January and May 2021. Forty-nine patients agreed to participate in the study. All participants gave their written informed consent and were >18 years of age at the follow-up examinations.

We divided the patients into three groups based on surgical procedure: Group 1 (*n* = 25; patients with no intraoperative sign of torsion = suspicion of spontaneous detorsion) had been treated with surgical exploration of the testis and bilateral orchidopexy to avoid a recurrent torsion of the testis; Group 2 (*n* = 20; patients with unequivocal torsion of the testis) had undergone surgical exploration of the testis, unilateral detorsion, and bilateral orchidopexy; Group 3 (*n* = 4; patients with necrotic testis) had undergone surgical exploration of the testis, unilateral orchiectomy, and contralateral orchidopexy.

All patients received a standardized medical examination, including inquiry of their medical history (family planning, body mass index [BMI], smoking status, medication, drug and alcohol consumption, symptoms in the operated testis, activity at the onset of TT symptoms, family history, physical activity and status). The International Index of Erectile Function (IIEF-5) questionnaire was used to determine sexual function. The physical examination included a scrotal ultrasound investigation, a blood test, and a spermiogram with an assessment of routine seminal parameters according to the World Health Organization (WHO), determination of OS, and anti-sperm antibodies (ASA).

The patients’ physical status was assessed on a scale from 1 to 5 (1: unfit, 5: fit). According to their COVID-19 status, patients were divided into two groups: the first had had a COVID-19 infection proven by a polymerase chain reaction test, and the second had not. Clinical data, if available, were obtained from the hospital records.

A blood sample was taken between 7 a.m. and 9 a.m. to determine hormonal status. FSH, LH, testosterone, free-testosterone (fT), sexual hormone binding globulin (SHBG), albumin, prolactin, and thyroid-stimulating hormone (TSH) were measured in the hospital laboratory. The normal ranges for the blood test were as follows: FSH 1.5–12.4 mU/mL; LH 1.7–8.6 mU/mL; testosterone 2.49–8.36 ng/mL; fT 6.76–22.76 pg/mL; SHBG 18.30–54.10 nmol/L; albumin 3.4–5 g/dL; prolactin 86–324 µU/mL; TSH 0.5–4.20 mU/L.

Semen was collected in the hospital by masturbation after two to five days of sexual abstinence. All samples were analyzed within one hour after collection. Semen analysis was performed according to the WHO 2010 criteria. ASA was analyzed by the mixed antigen reaction test (MAR test). OS was determined with the Male infertility Oxidative System (MyOxSIS, AYTU BioScience, Englewood, CO, USA) [22]. The normal range for OS was <1.38 mV/10^6^ mL.

### 2.1. Ethics Approval

The consent of the local ethics committee was obtained (EK: 1152/2020).

### 2.2. Statistics

#### 2.2.1. Comparisons of Two Subgroups

All data of continuous variables were checked for normal distribution (test of normality: Kolmogorov–Smirnov with Lilliefors significance correction, type I error = 10%) and, in the case of normal distribution, also for variance heteroscedasticity (Levene’s test, type I error 5%). In the case of normality and variance homogeneity, the independent two-sample *t*-test was used for group comparisons. In the case of normality but no variance homogeneity, Welch’s t-test was applied. For variables without normally distributed data and for variables measured on ordinal scales, the Mann–Whitney U test was used. Dichotomous variables were compared by Fisher’s exact test, and the remaining categorical variables by the chi-squared test.

#### 2.2.2. Comparisons of More Than Two Subgroups

As no continuous variables showed both normally distributed data and variance homogeneity, all continuous variables, as well as variables measured on ordinal scales, were compared by a non-parametric analysis of variance (Kruskal–Wallis test, followed by Nemenyi’s multiple comparisons). Data of categorical variables were analyzed by the chi-squared test, with the provision of adjusted residuals.

Correlations were reviewed by Bravais–Pearson correlation coefficients, Spearman’s rank correlation coefficients, and point biserial Spearman’s rank coefficients. Associations of continuous variables and variables measured on ordinal scales with categorical variables were investigated by eta coefficients (combined with analyses of variance using the categorical variable as a grouping variable—for details see above). Associations of dichotomous variables with categorical variables were investigated by Cramer’s V (combined with the chi-squared test and Fisher’s exact test). The influence of various parameters on oxidative stress was investigated by multiple regression analysis. The type I error was not adjusted for multiple testing. Therefore, the results of inferential statistics are only descriptive. Statistical analyses were performed using the open-source R statistical software package, version 4.0.5 (The R Foundation for Statistical Computing, Vienna, Austria). The detailed statistical analysis can be obtained from the authors on request.

## 3. Results

### 3.1. Demographic Data—Descriptive Statistics

Demographic data are shown in Table 1 and Table 2. The mean duration of follow-up was 101 months (1–253). None of the patients had a recurrent TT. Twenty-seven percent of the patients (13/49) reported symptoms in the testis after surgery, such as pain (*n* = 7), hematospermia (*n* = 1), hypersensitivity (*n* = 3), and a high-riding testis (*n* = 2); 57.1% of the patients (*n* = 28/49) were physically active and 96% of the patients (*n* = 47/49) felt fit.

#### 3.1.1. Family Planning

Ten patients (20.4%) had fathered a child after TT in ten cases. Assisted reproduction was not needed. One patient who was normozoospermic after TT reported a spontaneous abortion.

#### 3.1.2. Erectile Function

One patient reported moderate (Group 1), one patient mild to moderate (Group 1), and six patients mild erectile dysfunction (Group 1 three, Group 2 two patients, and Group 3 one patient), according to the IIEF-5 questionnaire. Three patients reported not having sexual intercourse.

#### 3.1.3. Incidental Findings during the Physical Examination

On physical examination, six patients had a hydrocele, six a spermatocele, one relative phimosis, and seven a varicocele (five subclinical, one Grade I, and one Grade II). Testicular microlithiasis was seen in two cases.

#### 3.1.4. Laboratory Parameters

Laboratory parameters are summarized in Table 2. LH was elevated in five cases (two cases in Group 1 and Group 2, respectively, and one in Group 3). In all of these cases, testosterone was normal. fT was slightly decreased in one of these cases (6.5 pg/mL; Group 2). All other patients had a normal fT. Two patients had decreased testosterone levels (2.46 ng/mL in Group 1 and 1.56 ng/mL in Group 2) with normal LH and fT. FSH was elevated in one case (13.9 mU/mL; Group 3). Elevated prolactin was seen in 23 cases (11 in Group 1 and 12 in Group 2). Three patients had elevated TSH levels. In two cases the levels were marginally elevated: one patient did not take any medication and one patient took thyroid medication. One patient had leukocytosis with an incidental finding of chronic lymphoid leukemia and was referred to the oncology department.

#### 3.1.5. Semen Parameters

Semen analysis was feasible in 48 cases (Table 2 and Table 3). One patient in Group 2 was unable to give a sample. Based on sperm quality, patients were divided into the following categories: normozoospermia with normal semen parameters, asthenozoospermia with reduced motility (progressive motile sperm <32%), and oligozoospermia (<15 × 10^6^/mL sperm count). Forty-two patients (87.6%) had normozoospermia, five (10.4%) oligozoospermia, and one (2.1%) asthenozoospermia. Three patients (6.3%) with normozoospermia had reduced vitality (one in Group 1 and two in Group 2); morphology was normal in all cases. MAR positivity was seen in four cases, all in Group 1. Agglutinate was detected in 48% of Group 1 (+8/25; ++4/16), 36.9% of Group 2 (+4/19; ++3/16), and 25% of Group 3 (+1/4). Five patients in Group 1, three in Group 2, and one in Group 3 had elevated OS. OS could not be evaluated in two cases (Group 2). The three groups did not differ significantly in regard to spermiogram categories (*p* = 0.183; Kruskal–Wallis test).

### 3.2. Comparison of Exploration, Detorsion, and Orchiectomy

**Figure 1:**
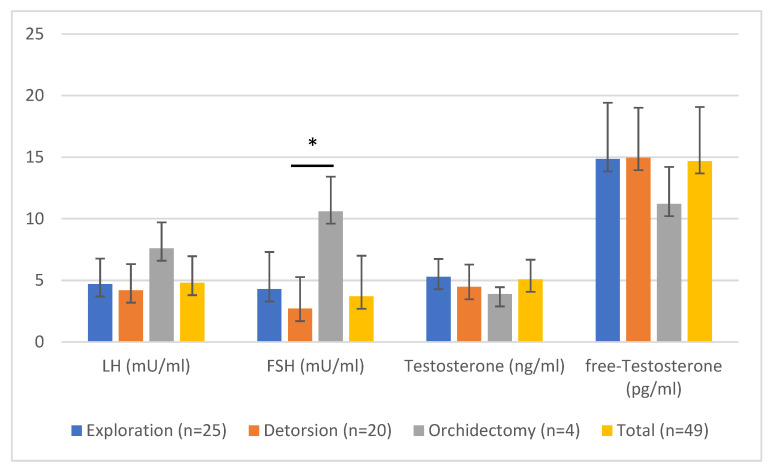
Laboratory parameters at control: LH, testosterone and free testosterone did not differ significantly between groups, whereas FSH was significantly elevated in patients with orchiectomy compared to those who underwent detorsion (* *p* = 0.005) but not exploration.

**Figure 2:**
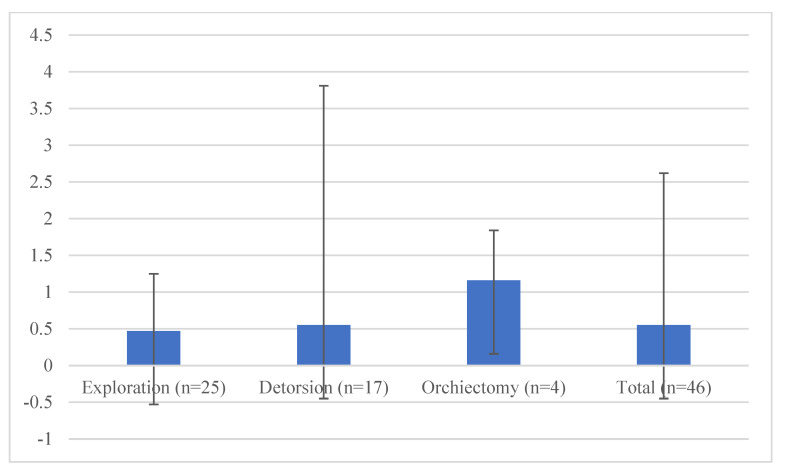
Analysis of oxidative stress (OS) in relation to the surgery performed.

Significant differences were seen between the three subgroups in the time from the onset of symptoms to surgery (*p* < 0.001), FSH (*p* = 0.003), round cell count (*p* = 0.002), and leukocytes in the spermiogram (*p* = 0.040) (Table 4 and Table 5). The grade of torsion did not differ significantly between Group 2 and Group 3 (*p* = 0.754). Patients with orchiectomy had elevated FSH and lower testosterone and free-testosterone levels compared to patients with preserved testes (Figure 1). OS did not differ significantly between the three groups (*p* = 0.764 Kruskal–Wallis test; Figure 2). A decreased sperm count, with normal morphology, was seen in Group 3 compared to Groups 1 and 2. However, vitality was improved in the orchiectomy group (Table 2). No significant difference was observed between groups in regard of erectile function (*p* = 0.563, Kruskal–Wallis test).

The analysis of OS (mV/10^6^ mL) was feasible in 46 cases. One patient could not give a semen sample and the analysis of OS was not applicable in two cases (all three patients in Group 2). There was no significant difference between the three groups (*p* = 0.764 Kruskal–Wallis).

#### Correlation Analysis

The results of the analysis are shown in Table 6; only the significant correlations regarding the investigated parameters and OS, erectile function, and the size of the affected testis are mentioned. Sperm morphology was negatively correlated with the time between surgery and follow-up (−0.367; *p* = 0.010 – Spearman’s test). A multivariate analysis of oxidative stress showed a significant correlation for oligozoospermia and normozoospermia (*p* = 0.002; beta regression coefficient 4.335). Vitality revealed a non-significant negative correlation with OS (−0.249; *p* = 0.096). A history of COVID-19 infection showed no significant association with OS and no relevant association with any investigated parameter.

## 4. Discussion

TT primarily affects young men who desire to father children [1,2]. Infertility concerns are rising in this specific population. However, a previous study revealed TT as a potential cause of infertility in only 0.5% of patients [23].

The time from the onset of symptoms to surgery, as well as the grade of torsion, are major factors affecting testicular viability [24]. Assuming that surgery is performed 25 to 48 h after the onset of symptoms, only 24.4% of testes can be preserved, while the number increases to 97.2% when surgery is performed within six hours of the onset of symptoms [25]. Accordingly, we found a significantly longer interval from the onset of symptoms to surgery between Group 2 and Group 3, as well as between Group 1 and Group 2. Interestingly, there was no significant difference between Groups 1 and 3. This may be due to an intermittent torsion. A significant negative correlation was noted between the preservation of the testis and the time from the onset of symptoms to surgery. Yang et al. [26] showed a mean grade of 360° of torsion in cases of detorsion, and 540° in patients treated with orchiectomy. We observed the same degrees of torsion in Groups 2 and 3. Furthermore, we made a distinction between patients who only underwent exploration and orchidopexy, and those who underwent detorsion of the testes. According to previous data, up to 32.5% of patients with an acute scrotum had a spontaneous detorsion of the testis during surgery [27]. A limitation of the present study is the small number of patients investigated after orchiectomy (*n* = 4). Furthermore, we assumed that patients treated with exploration of the testis and orchidopexy had an intermittent torsion. The patient cohort may have had other reasons for an acute scrotum, such as epididymitis. However, these patients could serve as controls for comparison.

A possible genetic predisposition to TT has been reported in up to 10% of cases [28]. Of our patients, 8% had a positive family history of TT. The majority of our patients had been asleep or in a state of rest at the onset of symptoms. A recurrent TT is a rare event [29]. We registered no recurrence in our population. Additionally, 27% of the patients reported symptoms in the affected testis after surgery for TT, such as pain, hematospermia, increased sensitivity, and a high-riding testicle.

Barsch et al. [5] analyzed endocrine and exocrine function of the testes in 30 patients with a previous TT: one half of the patients with symptoms ≤ four hours before surgery had a normal spermiogram. Three patients with symptoms >24 h to surgery who underwent orchiectomy had a normal spermiogram, while patients with symptoms >24 h to surgery for preserving the testis presented with abnormal semen parameters. In the orchiectomy group, we found normozoospermia in all cases but with lower total sperm counts and sperm concentrations compared with detorsion or exploration of the testes. Four patients in Group 2 and one in Group 1 had oligozoospermia.

Lower testicular volume is associated with decreased sperm parameters in patients [30,31]. Furthermore, infertile men have been revealed to have smaller testicular volume when compared to fertile men [32]. In our population, we demonstrated a significant positive correlation between the size of the affected testis and sperm concentration and total sperm count.

Diverse ASA data have been registered after TT [20]. Arap et al. [4] reported no significant difference in ASA between orchiectomy (21%, *p* = 0.073) or orchidopexy (20%, *p* = 0.17) compared to controls (14.5%). We noted MAR positivity in four cases; all patients had only undergone exploration of the testes. According to experimental animal studies, bilateral testicular damage and reduced fertility may occur after unilateral TT due to damage to the BTB, occurring only if the previously torqued testicle is left in place [6]. In our population, patients with detorsion had significantly higher round cell counts on their spermiogram compared to patients who had undergone exploration or orchiectomy. Approximately 90% of the round cells are normally sperm precursors [33], so a torsion of the testis might increase the slough off of sperm precursors. Thus, elevated round cell counts could be an indirect sign of testicular injury in patients with the testis left in place.

Late hormonal function may be affected after TT. A study comprising 12 patients with detorsion and orchidopexy and eight patients with orchiectomy revealed normal FSH, LH, and testosterone levels for all patients. However, inhibin B levels were significantly reduced in patients with torsion compared to controls [15]. Arap et al. [4] reported that patients with orchiectomy had significantly higher FSH and LH levels than patients with orchidopexy. Our study confirms these findings for FSH; LH was elevated after orchiectomy compared to orchidopexy. However, the difference was not statistically significant. We found patients with elevated LH in all three groups, but the majority of them had normal testosterone levels. Only one patient had reduced fT and elevated LH levels. Two patients had reduced testosterone, but normal LH and fT levels.

According to Zhan et al. [30], patients treated with orchiectomy had slightly, but not significantly, lower pregnancy rates and a longer mean time to pregnancy compared to patients treated with detorsion and orchidopexy (83.7%; 1.41 years +/− 0.71 vs. 91.3%; 0.74 +/− 0.11). Patients with TT in adulthood had lower pregnancy rates than those with TT in childhood. Patients treated with orchiectomy in adulthood had a significantly longer time to pregnancy in comparison with adults treated with orchidopexy [34]. A study investigating 63 patients with TT (41 orchidopexies and 22 orchiectomies) demonstrated no difference in pregnancy rates, and a slightly longer time to pregnancy in the orchiectomy group (90.2% vs. 90.9%; 6.6 +/− 5.50 vs. 7.2 +/− 5.4 months for orchidopexy vs. orchiectomy) [19]. In early life, TT appears to exert no significant effect on pregnancy rates. In our study, ten patients fathered a child after TT, and assisted reproduction was never needed. One patient with normozoospermia after TT reported a spontaneous abortion.

OS is considered one of the major factors in male infertility [10,11,12,13], and is associated with major semen parameters such as sperm concentration, total sperm count, and progressive motility. Our study is the first to investigate OS after TT. In accordance with previous findings [13,14], we noted a significant difference in OS between normozoospermia and oligozoospermia in a multivariate analysis. Furthermore, we found a significant positive correlation between OS and the patients’ height (0.337; *p* = 0.025), spermiogram category (0.337; *p* = 0.022), and medication history (0.32; *p* = 0.039), as well as a significant negative correlation between OS and age at control (−0.315; *p* = 0.033), sperm concentration (−0.573; *p* < 0.001), and total sperm count (−0.402; *p* = 0.006). We noticed a high variability in OS within the groups, which can be explained due to the dependence of OS from various clinical factors. Medication with antioxidants can improve semen quality through reducing OS [35]. However, several medications have been reported to have a negative impact on spermiogenesis, such as antidepressants, antibiotics, and antihypertensive agents [36]. Increased scrotal temperature due to fever or varicocele can cause a temporary decrease in sperm quality, where OS may play a role [37]. Smoking also results in elevated OS and decreased sperm count and morphology when compared with non-smokers [38]. Bacteriospermia, as a sign of infection, is accompanied by elevated OS and decreased fertility even in normozoospermic patients [39]. We could identify the above mentioned correlations with OS. However, due to the relatively low number of patients, the influence of other factors on OS cannot be ruled out. We registered no significant difference in OS between the three TT groups, possibly due to the small number of orchiectomy patients.

Despite the fact that TT is an absolute emergency in urological care and surgery is the gold standard of therapy, counselling patients prior to surgery is of great importance. However, due to the low incidence of TT as cause of infertility [4], the patients can also be reassured about the negative effects of the disease and surgery on exocrine and endocrine testicular function based on our data. In our study, no assisted reproduction was needed in case of family planning, as mentioned above. The role of time from the onset of symptoms to surgery, as well as the grade of torsion in testicular survivability, should be discussed with the patients.

## 5. Conclusions

The interval from the onset of symptoms to surgery and the degree of torsion are major factors influencing the viability of the testes. Erectile function, testosterone, and OS were not significantly affected by TT in the long-term follow-up. Orchiectomy results in elevated FSH and lower round cell counts compared to preservation of the testis. OS is significantly associated with sperm concentration, total sperm count, spermiogram category, medication history, age at follow-up, and the height of patients.

## Figures and Tables

**Table 1:** Demographic data of patients.

		Exploration Group 1		Detorsion Group 2		Orchiectomy Group 3		Total	
Investigated Parameter		*n*	%	*n*	%	*n*	%	*n*	%
Side of the affected testis	Left	10	40	7	35	0	0	17	35
	Right	15	60	13	65	4	100	32	65
	Total	25	100	20	100	4	100	49	100
Activity at the onset of Symptoms	Resting	9	42.9	3	25	1	33.3	13	36
	Sleeping	8	38.1	7	58.3	1	33.3	16	44
	Doing sports	4	19	2	16.7	1	33.3	7	19
	Total	21	100	12	100	3	100	36 ^b^	100
Undescended testis in the hashish	No	21	84	19	95	3	75	43	88
	Yes	4	16	1	5	1	25	6	12
	Total	25	100	20	100	4	100	49	100
Positive family history of TT ^a^	No	23	92	18	90	4	100	45	92
	Yes	2	8	2	10	0	0	4	8.2
	Total	25	100	20	100	4	100	49	100
Smoking	No	13	52	16	80	3	75	32	65
	Yes	12	48	4	20	1	25	17	35
	Total	25	100	20	100	4	100	49	100
Alcohol Consumption	No	8	32	9	45	1	25	18	37
	Yes	17	68	11	55	3	75	31	64
	Total	25	100	20	100	4	100	49	100

^a^ testicular torsion; ^b^ 13 patients were unable to recall what they had been doing at the onset of symptoms.

**Table 2:** Clinical data and laboratory parameters.

		Exploration (*n* = 26) Group 1		Detorsion (*n* = 19) Group 2		Orchiectomy (*n* = 4) Group 3		Total (*n* = 49)	
		Mean	SD ^a^	Mean	SD	Mean	SD	Mean	SD
Medical History	Age at surgery (years)	21	7.42	18	4.24	15.5	8.54	19	6.46
	Age at control (years)	28	6.54	27	5.28	32.5	6.66	28	5.98
	Time from the onset of symptoms to surgery (hours)	8	11.93	3	1.71	50	37.9	5	15.34
	Follow-up (months)	83	66.25	106.5	66.6	137.5	98.83	101	69.08
	Height of patients (meter)	1.8	0.08	1.8	0.07	1.79	0.08	1.8	0.08
	Weight of patients (kg)	75	16.5	79	20.15	82	18.12	77.5	17.92
	BMI ^b^ (kg/m^2^)	24.02	4.52	23.41	6.36	25.64	8.65	23.42	5.64
	IIEF-5 ^c^	25	3.95	24	1.58	25	3.46	24.5	3.17
	Size of the affected testicle (mL)	18	4.26	20	3.33	n.a.	n.a.	20	3.91
	Size of the unaffected testicle (mL)	20	4.51	20	4.09	16.5	3.5	20	4.29
	Grade of torsion (°)	0	0	360	207.04	540	236.7	0	241.61
	Fitness scale from 1 to 5 (the best is 5)	4	0.94	4	0.74	4.5	0.96	4	0.86
Laboratory parameters	FSH ^d^ (mU/mL)	4.3	3.01	2.7	2.57	10.6	2.83	3.7	3.31
	LH ^e^ (mU/mL)	4.7	2.07	4.2	2.13	7.6	2.12	4.8	2.17
	Testosterone (ng/mL)	5.29	1.45	4.47	1.82	3.89	0.56	5.08	1.6
	Free testosterone (pg/mL)	14.85	4.57	14.95	4.07	11.21	3.01	14.68	4.4
	Prolactin (µU/mL)	304	125.73	331	132.17	194	87.09	300	129.14
	SHBG ^f^ (nmol/L)	34	13.98	30.4	17.65	32.3	11.03	31.6	15.13
	Albumin (g/dL)	4.6	0.24	4.7	0.19	4.45	0.17	4.6	0.22
	TSH ^g^ (mU/L)	1.88	2.68	2.02	0.82	2.15	0.85	1.88	2
Semen analysis ^h^	Sperm concentration (million/mL)	52	45.35	50.2	70.87	34	10.88	50.7	56.05
	Total sperm count (million)	158.88	238.47	235.44	296.71	159.35	15.48	172.5	255.45
	Vitality (%)	68	10.12	65	14.52	70.5	2.06	68	11.99
	Progressive motility (a+b) (%)	53	8.01	55	13.23	55	18.78	54.5	11.17
	Overall motility (a+b+c+) (%)	65	7.62	62	11.57	66.5	13.67	64.5	9.88
	Fast progressive motility (a; %)	19	12.24	19	13.88	22	18.57	19	13.29
	Slow progressive motility (b; %)	35	12.65	31	10.99	26.5	12.07	31.5	11.92
	Non-progressive motility (c; %)	8	5.93	8	6.3	11.5	5.8	8.5	5.98
	Immotility (d; %)	35	7.7	38	11.57	33.5	13.67	35.5	9.92
	Semen pH	7.5	0.23	7.5	0.2	7.65	0.35	7.5	0.23
	Morphology (%)	11	6.85	12	4.83	12	4.9	11	5.94
	Round cell count (million/mL)	1	4.02	2.4	15.01	0.7	0.43	1.8	10.04
	Leukocytes (million)	0	0.36	0.2	0.94	0	0	0.05	0.66
	Oxidative stress ^i^ (mV/10^6^ mL)	0.47	0.78	0.55	3.26	1.16	0.68	0.55	2.07

^a^ standard deviation; ^b^ body mass index; ^c^ International Index of Erectile Function; ^d^ follicle-stimulating hormone; ^e^ luteinizing hormone; ^f^ sexual hormone binding globulin; ^g^ thyroid-stimulating hormone; ^h^ according to WHO 2010; ^i^ measured with the Male infertility Oxidative System (MyOxSIS).

**Table 3:** Semen analysis according to spermiogram categories.

	Exploration Group 1		Detorsion Group 2		Orchiectomy Group 3		Total	
	N	%	*n*	%	*n*	%	*n*	%
Normozoospermia	23	92	15	78.9	4	100	42	87.5
Oligozoospermia	1	4	4	21.1	0	0	5	10.4
Asthenozoospermia	1	4	0	0	0	0	1	2.1
Total	25	100	19	100	4	100	48	100

**Table 4:** Comparison of exploration, detorsion and orchiectomy I.

Investigated Parameter	Test	Overall *p*-Value
**Time from the onset of symptoms to surgery (hours)**	**KW**	**<0.001**
**FSH ^a^ (mU/mL)**	**KW**	**0.003**
**Round cell count (million/mL)**	**KW**	**0.002**
**Leukocytes (million)**	**KW**	**0.040**
Age at surgery (years)	KW	0.167
Age at control (years)	KW	0.704
Follow-up (months)	KW	0.441
Height of the patient (m)	KW	0.455
Weight of the patient (kg)	KW	0.535
BMI ^b^ (kg/m^2^)	KW	0.973
IIEF-5 ^c^	KW	0.985
Size of the unaffected testicle (mL)	KW	0.318
Fitness scale from 1 to 5 (5 denotes the best)	KW	0.691
LH ^d^ (mU/mL)	KW	0.094
Testosterone (ng/mL)	KW	0.073
Free testosterone (pg/mL)	KW	0.123
Prolactin (µU/mL)	KW	0.092
SHBG ^e^ (nmol/L)	KW	0.737
Albumin (g/dL)	KW	0.084
TSH ^f^ (mU/L)	KW	0.895
Sperm concentration (million/mL)	KW	0.466
Total sperm count (million)	KW	0.593
Vitality (%)	KW	0.239
Progressive motility (a+b) (%)	KW	0.885
Overall motility (a+b+c+) (%)	KW	0.465
Fast progressive motility (a; %)	KW	0.798
Slow progressive motility (b; %)	KW	0.391
Non-progressive motility (c; %)	KW	0.559
Immotility (d; %)	KW	0.455
Semen-pH	KW	0.385
Morphology (%)	KW	0.773
Oxidative stress ^g^ (mV/10^6^ mL)	KW	0.764
Spermiogram categories	KW	0.183
Side of the affected testis	Chi	0.356
Children fathered after testicular torsion	Chi	0.453
Smoking (yes/no)	Chi	0.126
Alcohol consumption (yes/no)	Chi	0.604
Medication history (yes/no)	Chi	0.399
Drug use (yes/no)	Chi	0.464
Symptoms in the operated testis	Chi	0.884
**Sports activities (yes/no)**	**Chi**	**0.026**
Family history of testicular torsion	Chi	>0.999
Fitness status (yes or no)	Chi	0.575
Activity at the onset of symptoms	Chi	0.807

The Kruskal–Wallis (KW) test or the chi test was used for statistical analysis. ^a^ follicle-stimulating hormone; ^b^ body mass index; ^c^ International Index of Erectile Function; ^d^ luteinizing hormone; ^e^ sexual hormone binding globulin; ^f^ thyroid-stimulating hormone; ^g^ measured with the Male infertility Oxidative System (MyOxSIS).

**Table 5:** Comparison of exploration, detorsion and orchiectomy II by Nemenyi’s multiple comparisons.

Investigated Parameter	Overall *p*-Value	Exploration vs. Detorsion	Exploration vs. Orchiectomy	Detorsion vs.Orchiectomy
Time from the onset of symptoms to surgery	<0.001 **	<0.001 **	0.831	0.048 *
FSH ^a^ (mU/mL)	0.003 **	0.156	0.08	0.005 **
Round cell count (million/mL)	0.002 **	0.013 *	0.49	0.019 *
Leukocytes (million)	0.040 *	0.296	0.399	0.091

The Kruskal–Wallis test, followed by Nemenyi’s multiple comparisons, were used for statistical analysis. *, ** Means and standard deviations are listed in Table 1. ^a^ follicle-stimulating hormone.

**Table 6:** Analysis of correlations between oxidative stress (OS), erectile function, size of the affected testis, and the investigated parameter.

	Parameter	Correlations Coefficient	*p*	*n*	Test	*p*	Eta-Coefficient ŋ^2^
Oxidative stress ^a^	Age at control (years)	−0.315	0.033	46			
	Height of patient (m)	0.337	0.025	44			
	Sperm concentration (million/mL)	−0.573	<0.001	46			
	Total sperm count (million)	−0.402	0.006	46			
	Spermiogram category	0.337	0.022	46			
	Medication history	0.32	0.039	42			
Erectile function ^b^	Weight of the patient (kg)	0.364	0.015	44			
	BMI (kg/m^2^)	0.3	0.048	44			
	Physical status	0.439	0.002	46			
	Sports activities	0.301	0.042	46			
Size of the affected testicle	Size of the normal testicle (mL)	0.755	<0.001	44			
	Sperm concentration (million/mL)	0.391	0.009	44			
	Total sperm count (million)	0.416	0.005	44			
	Round cell count (million/mL)	0.391	0.009	44			
	Children after torsion	0.566	0.035	14			
	Medication history	−0.324	0.039	41			
	Activity at the onset of symptoms	-	-	-	KW	0.047	0.267

BMI: body mass index; FSH: follicle-stimulating hormone; KW: Kruskal–Wallis. Only significant results are shown; the detailed statistical data can be obtained from the authors on request. ^a^ Oxidative stress analysis was feasible in 46 cases; ^b^ according to the IIEF-5 questionnaire.

## Abbreviations

ASA	Anti-sperm antibodies
BMI	Body mass index
BTB	Blood-testis barrier
FSH	Follicle-stimulating hormone
fT	Free testosterone
IIEF-5	International Index of Erectile Function
LH	Luteinizing hormone
MAR-test	Mixed antigen reaction test
MyOxSIS	Male infertility Oxidative System
OS	Oxidative stress
ROS	Reactive oxygen species
SD	Standard deviation
TT	Testicular torsion
WHO	World Health Organization
